# The Relationship Between (Endo)cannabinoids, the Microbiome and Melanoma

**DOI:** 10.1002/prp2.70304

**Published:** 2026-07-31

**Authors:** Farideh A. Javid, Marin Golčić, Anja Harej Hrkać, Andrej Belančić

**Affiliations:** ^1^ Department of Pharmacy, School of Applied Sciences University of Huddersfield Huddersfield UK; ^2^ Clinic for Tumors Clinical Hospital Centre Rijeka Rijeka Croatia; ^3^ Department of Basic and Clinical Pharmacology With Toxicology Faculty of Medicine, University of Rijeka Rijeka Croatia

**Keywords:** cannabinoids, gut microbiome, melanoma, skin cancer

## Abstract

Despite advances in targeted and immunotherapy for melanoma, resistance to treatment and severe adverse effects pose significant challenges. This necessitates the development of new treatment strategies, and cannabinoids, because of the extensive preclinical evidence for their cytotoxic action on carcinoma cells in a variety of cancers, can offer a novel therapeutic option when used appropriately. Indeed, the potential therapeutic benefits of cannabinoids were formally recognized in the world and much more recently in the United Kingdom when cannabis‐based medicinal products were moved from Schedule 1 of the Misuse of Drugs Regulations 2001 to Schedule 2 in 2018. This move further encouraged scientists to look at more applications of cannabinoids in different disorders. Although the potential psychoactivity of cannabis as a Schedule 1 drug hinders research, more research could focus on non‐psychoactive components such as cannabidiol (CBD) and cannabigerol (CBG). This review summarizes some past and current research on the relationship between the cannabinoid system and the microbiome in patients with metastatic melanoma who undergo immunotherapy. The review also provides a comprehensive background on the function of the cannabinoid system in normal and diseased skin, as well as future directions in using cannabinoids as an adjunct to chemotherapeutics in the treatment of the disease.

## Graphical abstract

CB_1_ and CB_2_ receptors are widely distributed in both normal and cancerous cells. Cannabinoids have a positive impact on reducing tumor growth in mice. A positive relationship exists between CB_2_ receptors and survival in skin melanoma. However, there is a negative impact on non‐small cell lung cancer and colon cancer, whereas a higher association of CB_1_ receptors is linked to longer survival in uveal melanoma. The role of CB receptors may vary depending on the type of cancer. The effect of cannabinoids appears to be dose‐dependent, with lower concentrations promoting cancer proliferation, while higher concentrations induce apoptosis in cancer cell lines. Furthermore, there is a significant and bidirectional relationship between the gut microbiome and cannabinoids.

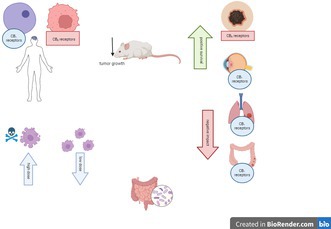

## 1 Melanoma

Melanoma is a cancer that originates from melanocytes, most commonly in the skin, although there are at least nine distinct entities of melanoma, including uveal and mucosal melanoma [1]. Its incidence and mortality rates differ widely throughout the world, with the highest incidence observed in Australia and New Zealand and the lowest in Asian and African countries. The most common sites of metastasis include the liver, bone, and brain, while for patients with local melanoma, more than half of all recurrences/metastases occurred within 3 years [2]. Advanced melanoma has become a new oncological model for solid cancer treatment due to improved biological understanding and availability of novel therapeutic agents [3, 4].

At the beginning of the 21st century, the median survival of metastatic melanoma was only about 6 months, with a 5‐year survival of only 6% [5]. However, earlier diagnostics and improved treatment options have resulted in a 5‐year relative survival rate of 94% for all stages of melanoma in the most recent analysis [6]. The significant improvement in the metastatic stage of melanoma was due to the discovery of checkpoint inhibitors, including anti–programmed cell death protein 1 (anti–PD‐1) and anti–cytotoxic T‐lymphocyte–associated protein 4 (anti–CTLA‐4) immunotherapy, as well as targeted BRAF (B‐Raf proto‐oncogene, serine/threonine kinase)/MEK (mitogen‐activated protein kinase kinase) inhibitors. Compared to chemotherapy, which was a previous standard of treatment, nivolumab, an anti‐PD‐1 antibody, achieved 72.9% 1‐year survival compared to 42.1% in the chemotherapy group (HR 0.42, 99.79% CI, 0.25–0.73; *p* < 0.001) [7]. Combining anti‐PD‐1 and anti‐CTLA4 immunotherapy resulted in even more impressive survival: a median overall survival (OS) of 72.1 months and a 6.5‐year OS of 46%–57%, depending on the presence of BRAF mutations [8]. Newer data also showed the potential use of combining the newer anti‐Lymphocyte activation gene 3 antibody with anti‐PD1, resulting in longer progression‐free survival at 12 months compared to anti‐PD1 monotherapy (47.7% (95% CI, 41.8–53.2) vs. 36.0% (95% CI, 30.5–41.6)). However, the combination was not tested against the combination of anti‐CTLA‐4 and anti‐PD1 [9].

Although BRAF/MEK inhibitors also achieved significant responses in the metastatic stage, a recent phase III trial (DREAMseq) demonstrated that the use of dual anti‐PD‐1 and anti‐CTLA‐4 immunotherapy achieved even greater survival compared to BRAF/MEK inhibitors in patients with BRAF mutants in the first line of therapy (2‐year OS 71.8% vs. 51.5%, *p* = 0.010). Therefore, immunotherapy is the recommended first‐line choice for most patients [10].

Despite improvements in 5‐year survival of metastatic melanoma from 6% to more than 50% in less than 50 years [11], the most recent data demonstrate that only 43% of the study population is alive after 10 years of follow‐up, and more than 2/3 of patients have progressed [12]. Therefore, while targeted treatment and immunotherapy offered a revolutionary step toward a cure, they are not sufficient to achieve a cure even in half of the patients, and there are still significant unmet needs. Additionally, predictive and prognostic factors/biomarkers for the response rate, as well as disease progression, remain an open question and a topic worth investigating. Several factors are associated with a poor prognosis in immunotherapy‐treated patients and include elevated levels of C‐reactive protein and lactate dehydrogenase, loss of human leukocyte antigen (HLA) class I expression in melanoma cells which was in turn associated with reduced T cell infiltration and an environment enriched with myeloid suppressor cells, low levels of interferon (IFN)‐gamma, and high tumor burden.

Some of the most novel and potentially promising factors include cannabis and the signature probiotics of the gut microbiome. Although the expanding body of literature is still relatively scarce due to the potential importance and novelty of these two factors, our objective was to review the body of evidence and comment on the relationship between the cannabinoid system and the gut microbiome in metastatic melanoma while immunotherapy is implemented.

## 2 Cannabinoid System and Cannabinoid‐Based Medications

Although cannabis is illegal in most countries around the world, it is estimated that 26%–31% of cancer patients used cannabis after cancer diagnosis [13], with up to 40% of patients believing that it is useful for cancer treatment [14]. Numerous studies have been conducted on the effects of cannabinoids on preclinical models of diseases ranging from central nervous system (CNS) disorders to immune diseases. Some clinical trials have also been carried out that led to the introduction of some cannabis‐based drugs such as dronabinol, nabilone, nabiximol (sativex), and, more recently, epidiolex [15, 16, 17, 18]. In fact, the FDA has approved three cannabis‐related drug products and one cannabis‐derived drug product, only with a prescription from a qualified healthcare professional. These products include Epidiolex, which contains a purified form of the drug substance cannabidiol (CBD), for the treatment of seizures associated with Lennox‐Gastatut syndrome Dravet syndrome, or tuberous sclerosis complex, Marinol and Syndros [products with dronabinol, a synthetic delta‐9‐ tetrahydrocannabinol (THC)] for the treatment of weight loss in patients with acquired immunodeficiency syndrome, and Cesamet (nabilone) for the treatment of nausea and vomiting associated with cancer chemotherapy in patients who have not responded adequately to conventional antiemetic treatments [19].

The main psychoactive component of cannabis is THC, and other important nonpsychoactive components found in cannabis are CBD and cannabigerol (CBG) [20, 21]. Cannabinoids were found to affect specific receptors. CB_1_ receptors were the first to be identified in the brain in 1988 [22]. Later in 1993, a second receptor, CB_2_ receptors, was identified with high expression in B lymphocytes and natural killer cells, suggesting a possible role in the immune system [23, 24]. Endocannabinoids were identified to play a role in the modulation of pain, movement, feeding behavior, memory, mood, neuroprotection, and inflammatory responses and cancer [25, 26, 27, 28, 29, 30, 31, 32, 33, 34, 35, 36]. The endocannabinoid system (ECS) consists of endocannabinoid ligands such as anandamide and 2‐arachidonoylglycerol, and enzymes involved in the synthesis and metabolism of these lipid mediators, fatty acid amide hydrolyse (FAAH) and monoglyceride lipase (MAGL) [37, 38, 39], and receptors.

Both CB_1_ and CB_2_ receptors are G‐protein coupled receptors and the endocannabinoid, anandamide, can activate both. CB_1_ receptors are expressed mainly in the central nervous system, and their activation reduces gamma‐aminobutyric acid (GABA) release, which in turn leads to an increase in the level of dopamine (DA), which is responsible for the psychoactive and euphoric response. The release of glutamate is also reduced in the CNS after activation of CB_1_ receptors, which in turn protects against excitotoxicity induced by glutamate; it has highlighted the potential for use in neurological disorders [40, 41, 42, 43].

Cannabinoids also bind to noncannabinoid receptors such as peroxisome proliferator‐activated receptors—PPARs (PPARα and PPARγ receptors) and GPR55 (orphan G‐protein coupled receptor) and TRIPV1 (transient receptor potential channels such as transient receptor potential vanilloid 1) receptors [44, 45, 46, 47, 48]. All of the receptors mentioned above have also been present in different compartments of the skin and their activities exert potent effects, particularly under pathological (i.e., inflammatory) conditions.

## 3 Cannabinoid and Non‐Cannabinoid Receptors in the Human Skin

**FIGURE 1:**
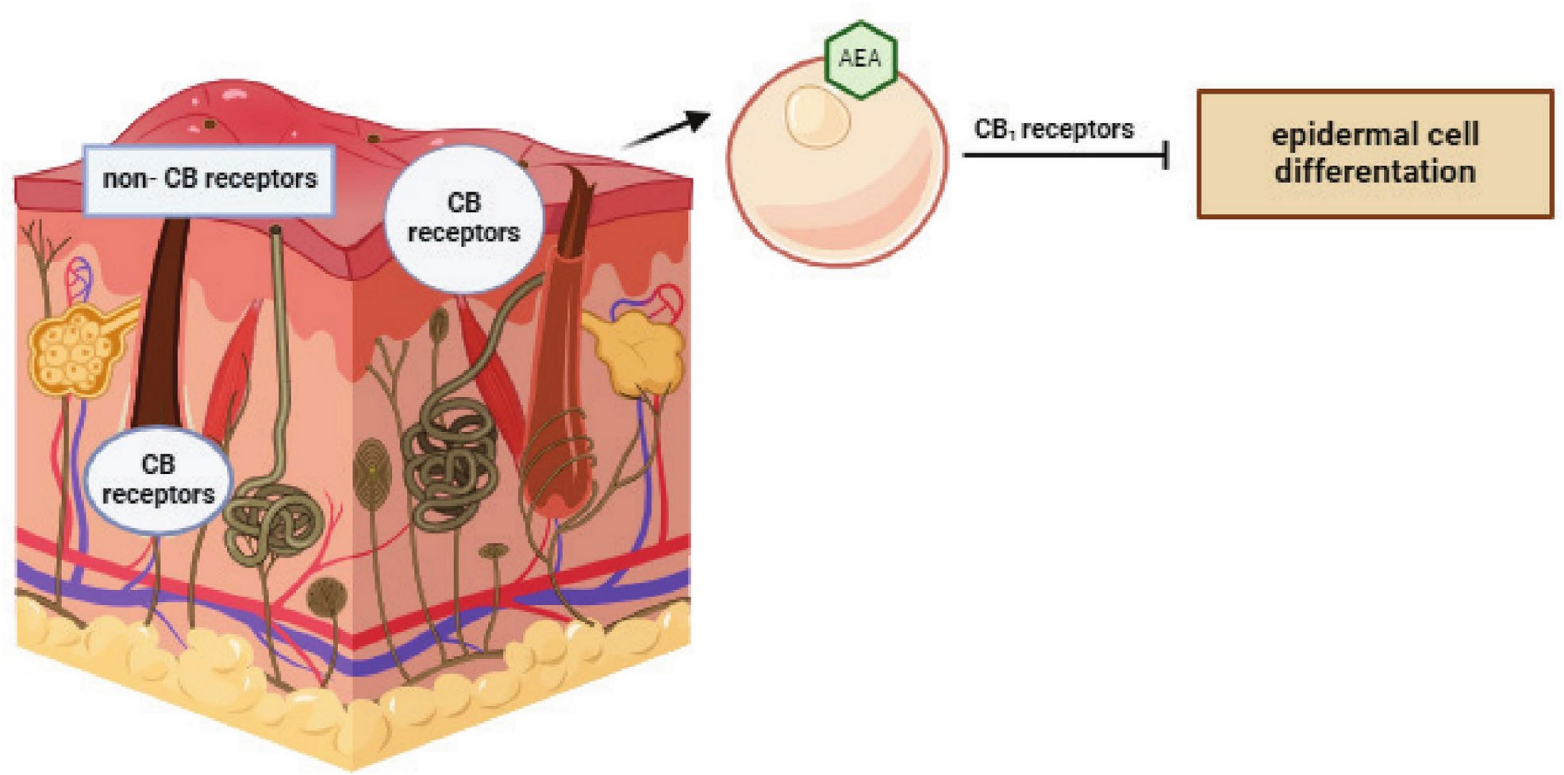
Cannabinoid and non‐cannabinoid receptors in human skin. Physiological functions of the endocannabinoid system. This figure illustrates the key components of the endocannabinoid system in human skin, highlighting CB and non‐CB receptors in different skin compartments, such as sebocytes derived from keratinocytes, epidermal, and sebaceous glands. Anandamide in human keratinocytes can induce an inhibition of epidermal cell differentiation by inhibiting protein kinase C activation via CB_1_ receptors.

The CB_1_ and CB_2_ receptors are expressed in the human skin (Figure 1). Cannabinoids have also been implicated in the normal physiology of the skin and also in the pathology of the skin. There has been evidence to indicate a functional role for CB_1_ and CB_2_ receptors, as well as non‐CB receptors such as TRIPV1 receptors [45, 48, 49]. This is in addition to studies showing the presence of endocannabinoids such as anandamide in human keratinocytes that induced an inhibition of epidermal cell differentiation by inhibiting protein kinase C activation via CB_1_ receptors [50]. Other studies also have shown that both CB and non‐CB receptors have been identified in different skin compartments such as keratinocytes, epidermal and sebaceous gland‐derived sebocytes [39, 51, 52, 53, 54, 55]. In fact, CB_1_ receptors have been found in sensory neurones of the skin, sebaceous glands, hair follicles, and immune cells, as well as differentiated keratinocytes [44]. Activation of CB_1_ receptors has been demonstrated to reduce pain and itch sensation, hair follicle growth, regulate keratinocyte proliferation and differentiation, and release of inflammatory mediators, and to control skin homeostasis [44, 56]. CB_1_ receptors were also suggested to be involved in epidermal cell differentiation, with a higher level of expression in more differentiated granular and spinous layers in an in situ model [53].

Activation of CB_2_ receptors was shown to promote sebum production, inhibition of the inflammatory response, proliferation, and differentiation of keratinocytes, as well as modulation of pain sensation [44, 56].

However, although limited, there is also conflicting evidence for the role of CB receptors in proliferation in the epidermal layer. For example, studies showed that pretreatment of transformed human keratinocytes, E6 and E7 oncoproteins of human papillomavirus type 16 (HPV‐16 E6/E7), with cannabinoid agonists and delta‐9‐THC inhibited cell growth independently of activation of the CB_1/2_ receptor [57]. Furthermore, studies by Casanova et al. indicated that both CB_1_ and CB_2_ receptor agonists did not affectcellular growth of human adult low‐calcium, high‐temperature keratinocytes (HaCaT), normal human epidermal keratinocytes (NHEK), and murine MCA3D keratinocytes [58]. However, the same group reported the involvement of the CB_1_ and CB_2_ receptors in mediating an inhibitory action on the growth of PDV.C57 and HaCaT cells (tumorigenic transformed murine keratinocytes). In such studies, the inhibitory action on cell growth provided by CB_1_ and CB_2_ receptor agonists was attenuated by CB_1_ and CB_2_ receptor antagonists. Further studies focused on the action of anandamide, an endocannabinoid, in keratinocytes and showed inhibition of cell proliferation and the apoptotic event afforded by anandamide through changes in mitochondrial membrane potential, Annexin‐V, and pro‐apoptotic caspases. At high concentrations, anandamide induces necrosis. All events were mediated by the CB_1_ and TRIPV1 receptors, followed by an increase in the level of Ca^2+^ influx sequentially [59]. The results were in line with previous experiments by Hermann et al. [60], who showed that CB_1_ receptor agonists significantly affected Ca2^+^ influx via an effect on TRIPV1 receptors through a cAMP coupling mechanism [60]. Further evidence for the sequential activity of the CB_1_ and TRIPV1 receptors came from studies in which mice deficient in the CB_1_ gene were used to show constitutive activity of the CB_1_ receptors necessary to maintain the activity of the TRIPV1 receptor channel in response to noxious chemical stimuli [61]. Therefore, previous studies provided evidence for a physiological role for endocannabinoids in the proliferation of human keratinocytes in the epidermal layer through CB_1_ and not CB_2_ receptors.

However, the above studies do not negate the role of CB_2_ receptors, as anandamide also has the affinity to bind to CB_2_ receptors [49, 62]. In this regard, studies have reported the involvement of CB_2_ receptors in mediating pain and inflammation in the skin. CB_2_ receptors were involved in alleviating pain by mediating the release of endogenous opioids involved in inhibiting pain in the skin afferent neurones [63]. Further studies of the involvement of CB_2_ in inflammation processes came from studies in which a significant increase in the level of endocannabinoids such as 2‐AG was reported after contact dermatitis in the ears of mice induced by oxazolone [39]. The swelling was significantly attenuated with the administration of a CB_2_ receptor antagonist, SR144528, and only marginally with a CB_1_ receptor antagonist, AM251 [64]. Further experiments by the same authors concluded the same results in chronic contact dermatitis.

Further experiments also showed that a reduction in allergic reaction in the skin following an increase in the level of endocannabinoids, and also deletion of both the CB_1_ and CB_2_ receptors or the CB_2_ receptors in mice induced a frequency of scratching that led to severe ulceration of the neck and head of the animals [39].

There is evidence to show a reduction in the level of expression of PPAR receptors in some inflammatory skin conditions such as psoriasis, allergic contact dermatitis, and atopic dermatitis. Studies have shown that activation of PPARα and PPARγ receptors reduces keratinocyte proliferation, leading to a reduction in epidermal hyperplasia [65]. This suggests that activation of these receptors by cannabinoid agonists has potential therapeutic action [47].

The involvement of TRIPV1 in mediating cannabinoid‐mediated effects is interesting and complex. Unlike 2‐AG, one of the main endocannabinoids that has a high affinity to activate only CB_2_ receptors, anandamide has an affinity not only to activate CB_1_ and CB_2_ receptors but also to activate TRIPV1 receptors. Anandamide is converted to ethanolamide and arachidonic acid by fatty acid amide hydrolase (FAAH), which in turn are oxidized by lipoxygenase enzymes to metabolites that activate TRIPV1 receptors [66]. Studies have shown that stimulation of TRIPV1 receptors by cannabinoids counteracts the effects that are caused by stimulation of the CB_1_ and CB_2_ receptors. It was an intriguing observation that endocannabinoids such as anandamide, when applied at low concentrations, reduced an effect mediated by TRIPV1 through the activation of CB_1_ receptors; however, in one study, anandamide at higher concentrations activated only TRIPV1 and not CB_1_ or CB_2_ receptors [59, 61]. It should be noted that cannabinoids can induce neurogenic inflammation, pain, and itch sensation by activating TRIPV1 [44]. Anandamide is produced by not only enzymes responsible for its synthesis and metabolism in the epidermis, hair follicles, and sebaceous glands. Therefore, it is not surprising that they are involved in the regulation of skin homeostasis [56].

G protein–coupled receptor 55 (GPR55) is expressed in keratinocytes and also in melanocytes. Studies have shown that endocannabinoids, through activation of GPR55 receptors, could attenuate both cell viability in melanocytes and pain hypersensitivity [67, 68]. It is known that in psoriasis, nerve growth factors play a significant role in stimulating keratinocyte proliferation. It was an interesting observation that GPR55 activation could reduce nerve growth factor, indicating a promising therapeutic approach to combat psoriasis [69].

## 4 Implication of the Cannabinoid System in Melanoma

Initial studies of cannabinoids in rodents and cell cultures were predominantly positive, demonstrating beneficial effects on overall survival in mice with melanoma and carcinoma cells compared to animals or cells that received chemotherapeutics without cannabinoid treatment [70, 71].

Armstrong et al. demonstrated a synergistic cytotoxic effect on melanoma cells with cannabidiol and THC through autophagy [72]. Another study using a CB_1_ agonist in severe combined immunodeficient mice with melanoma also showed a reduction in liver metastasis [73]. Similarly, Richtig et al. demonstrated that cannabinoids can significantly decrease tumor growth in vivo in mice through the CB_1_, TRPV1, and PPARα receptors, leading to cell death through caspase‐ mediated cell death. In further experiments, pretreatment with CB_1_ receptor antagonists that were applied before administration reversed the reduction in tumor growth, indicating the likely involvement of CB_1_ receptors [74]. Another recent study also showed that CBD reduced cell viability in melanoma cells and did not interfere with commonly used targeted therapy in metastatic melanoma [75]. While in vitro and in vivo studies highlighted the potential for the use of cannabinoids in the management of melanoma, more recent clinical investigations indicated that cannabinoids can interfere with the action of immunotherapeutic drugs such as immune checkpoint inhibitors, pembrolizumab, or nivolumab. This interaction could reduce the effectiveness of the treatment or exacerbate the adverse effects. For example, in a retrospective study by Taha et al. [76], it was found that in patients with melanoma who were treated with nivolumab plus cannabis, progression‐free survival and overall survival were not affected, and patients who consumed a higher THC content showed a better response rate [76]. Although cannabis use showed a detrimental effect on overall survival in the univariate analysis (*p* = 0.045, HR = 1.58, 95% CI 1.01–2.46), it did not affect progression‐free survival (PFS) or overall survival in the multivariate analysis. However, a prospective trial that included 34 patients with melanoma showed even worse results for cannabis use, with a hazard ratio (HR of 2.18, 95% CI 1.241–3.819) for overall survival (*p* = 0.007) [74].

In another prospective and observational study, the inclusion of cannabinoid plus immune checkpoint inhibitors in patients with metastatic malignancies induced a significant reduction in tumor progression time and overall survival time, although adverse effects such as hepatitis and arthritis, colitis, renal deficiencies, thyroid and skin toxicities were reduced [77]. This was followed by a reduction in the level of endocannabinoids. This was an interesting observation, as the level of endocannabinoids in the blood of cannabis users is higher compared to non‐cannabis users [78]. It is not clear how cannabinoids affect the responses to immunotherapy. Cannabinoids may affect the tumor microenvironment, and as a result, negatively modulate the effect of immunotherapy. Indeed, several studies have pointed to the tumor microenvironment as the main site of the action of cannabinoids. The application of THC inhibited the growth of melanoma through antagonistic effects primarily on its microenvironment [79]. Similarly, another study highlighted the protective role mediated by B cells of CB_2_ receptors in melanoma, showing that in mice deficient in the CB_2_ receptor, there was an abundance of largely undifferentiated B cells, leading to a poorer tumor‐specific immune response. The same study also showed a strong positive correlation between the expression of CB_2_ receptors and OS in skin melanoma [80]. A similar finding was observed for dendritic cell maturation in CB_2_ receptor‐ deficient mice [81].

In contrast, in a model of non‐small cell lung cancer, CB_2_ receptor deficiency in leukocytes resulted in a reduction in tumor burden with increased accumulation and tumoricidal activity of CD8+ T and natural killer cells and an improved response to anti‐PD1 immunotherapy [82].

At least some of the deleterious effects of cannabinoids were shown to be the result of the function of tumor‐specific T cells through CB_2_ receptors [83]. The above studies may indicate that the role of receptors in cancer settings depends on the type of cancer.

Another possible explanation for the interaction of cannabinoids with the effects of immunotherapeutic agents may involve the gut microbiome. The following sections review the evidence for such a possible interaction.

Although the above findings were important to note, limitations such as the unavailability of accurate/uniform dosages of cannabinoids and the advanced stage of the disease should be taken into account when interpreting the results.

## 5 Cannabis Extracts and Melanoma: Emerging Evidence

While it is easier to pharmacologically evaluate a single molecule, growing evidence suggests that complex cannabis extracts may exert additional biological effects through synergistic interactions between phytocannabinoids, terpenes, and other bioactive constituents.

Recently, studies have demonstrated the antiproliferative effects of a *Cannabis sativa* extract, PHEC‐66, on human melanoma cell lines [84]. PHEC‐66, which contains approximately 60% CBD, was evaluated in both 2D cell cultures and more physiologically relevant 3D spheroid models. The application of PHEC‐66 significantly reduced melanoma cell viability in a dose‐dependent manner across multiple cell lines, with a cytotoxic effect more pronounced in melanoma cells than in non‐cancerous skin cells, suggesting a degree of selectivity [84]. Further studies by the same group showed that the application of PHEC‐66 induced an upregulation of pro‐apoptotic markers (BAX) and a downregulation of anti‐apoptotic markers (Bcl‐2), induction of deoxyribonucleic acid (DNA) fragmentation, cell cycle arrest at the G1 phase, and a significant increase in intracellular reactive oxygen species (ROS) leading to cell death in melanoma [85]. Interestingly, while the authors showed that CB_1_ and CB_2_ receptors may play a role, since the cytotoxic effect of PHEC‐66 was diminished when cells were pretreated with the CB_1_ and CB_2_ antagonists, their exact functional contribution requires further investigation [85].

On the other hand, other recent studies investigated the immunomodulatory effects of CBG on melanoma, demonstrating, among other insights, the colony‐stimulating factor‐1 (CSF‐1) suppression as a novel target of cannabinoids [86]. Importantly, the authors showed that combining CBG with anti–PD‐L1 immunotherapy enhanced treatment efficacy, resulting in improved tumor control, survival, and increased infiltration of activated cytotoxic T cells compared to either treatment alone. However, studies in human patients remain necessary [86].

Other studies have also reported an antiproliferative effect associated with the use of *Cannabis sativa* leaf extracts when tested on multiple cancer cell lines, including melanoma, for [87], as well as an anti‐melanogenic effect for minor phytocannabinoids such as CBG, CBN, and CBC [88] and also for a combined hemp–ginger extract [89].

Taken together, these findings suggest that cannabis extracts may represent a promising adjunctive approach in melanoma treatment. Nevertheless, significant challenges remain, including variability in extract composition, lack of standardized dosing, and incomplete understanding of interactions with current therapies such as immune checkpoint inhibitors. Future studies, particularly well‐designed clinical trials, are required to clarify the therapeutic potential and safety profile of cannabis extracts in melanoma.

## 6 Gut Microbiome and Melanoma

Although controversial and heterogeneous results on the benefit of cannabinoids can be associated with the bimodal mechanism of action and variability of the presence of CB_1_ and CB_2_ receptors in different cancers and normal cells, the gut microbiome undoubtedly plays a role. The gut microbiome represents the genomic content of bacteria, archaea, and eukarya that colonize the gastrointestinal tract. The microbiome is responsible for a variety of beneficial effects on the host through a variety of physiological functions, but its dysbiosis can cause various diseases [90].

There is a growing body of research showing that the presence of certain types of bacteria can predict the response to immunotherapy [91, 92], also influencing the time necessary for a complete response to immunotherapy [93]. Although there is heterogeneity between the trials, we now know that patients with an abundance of the Ruminococcaceae family, Bifidobacteria, Faecalibacteria, Akkermansia, and Faecalibacteria, among others, are associated with an improved response to immunotherapy in the treatment of metastatic melanoma [94].

The gut microbiome was shown to be a prognostic factor, as modification of the gut microbiome with Bifidobacteria or fecal microbial transplantation was shown to increase the effectiveness of PD‐L1 immunotherapy [95]. Similar data were obtained for anti‐CTLA‐4 immunotherapy [96], both of which are the mainstay of melanoma treatment; however, the mechanisms responsible for this beneficial effect have not been fully elucidated. Identifying the mechanism of action is important, as up to 31% of cancer patients used unspecified probiotics during immunotherapy. However, another study showed that probiotic use was associated with a lower response rate to immunotherapy and potentially detrimental effects [97]. Therefore, it is not yet clear what the optimal microbiome is and what other factors influence its effectiveness. One of the most significant extrinsic factors that influence the microbiome includes diet [97], and baseline co‐medications such as glucocorticoids > 10 mg/day, proton pump inhibitors (PPIs), psychotropic drugs, morphine, and insulin were associated with significantly shorter overall survival [98, 99]. Although the use of antibiotics can decrease the effectiveness of immunotherapy [100], fecal microbial transplantation can improve response in human patients [101]. Therefore, there is a significant and bidirectional relationship between the microbiome and cannabinoids.

## 7 The Gut Microbiome that Influences the Cannabinoid System

The first evidence for the role of the microbiota in the regulation of intestinal endocannabinoid tone came from studies by Rousseaux et al., who demonstrated that oral administration of *Lactobacillus acidophilus* to mice and rats increased the expression of intestinal epithelial CB_2_ receptors, demonstrating the role of the intestinal microbiota in the regulation of intestinal endocannabinoid tone. By modifying the intestinal microbiota through various strategies, including antibiotic therapy, probiotic treatment, high‐fat diet (HFD), and mutations in the Myd88 gene that alter the toll‐like receptor (TLR)‐mediated bacteria‐host interaction, the significance of the intestinal microbiota in the regulation of intestinal endocannabinoid tone was further established. In the latter models, changes in the abundance of CB_1_ receptor messenger ribonucleic acid (mRNA) were observed in the colon and not in the jejunum [102, 103].

Similarly, deletion of the myeloid differentiation primary response gene 88, a central adapter molecule for most Toll‐like receptors (TLRs), changed intestinal regulatory T cells, intestinal peptide expression, and anti‐inflammatory endocannabinoids. Importantly, this protective effect was shown to be transferable by gut microbiota transplantation [104].

Another study also showed that the expression of different receptors in the duodenum, including CB_1_ receptors, depended on whether the mice were germ‐free or conventionally raised. Furthermore, the causal relationship was proven, as fecal microbiota transplantation (FMT) from conventionally raised mice to germ‐free mice reversed several alterations that affected the endocannabinoid system [105].

Lactobacillus strains were shown to induce the expression of intestinal epithelial CB_2_ receptors [102]. In the murine model of colon cancer, the same strain improved the response to antitumor immunotherapy [106].

*Akkermansia municiphila* can also increase intestinal levels of endocannabinoids, leading to increased inflammation control [107]. *Akkermansia municiphila* is, on the other hand, also one of the most researched bacterial strains associated with an improved response to checkpoint inhibitor immunotherapy [108, 109].

Changing the microbiome through diet can also lead to a change in endocannabinoids, as low vitamin D intake induced a lower microbial diversity characterized by an increase in Firmicutes and a decrease in Verrucomicrobia and Bacteroidetes, resulting in a change in endocannabinoid levels in the gut [110].

Similarly, the Mediterranean diet increased *A. municiphila* and changed the level of endocannabinoids in plasma [111].

The intestinal microbiota was shown to control the expression of CB_1_ receptors, which in turn control intestinal permeability. Blocking CB_1_ receptors with selective antagonists improved intestinal permeability, while activation of CB_1_ increased intestinal permeability [112].

However, increased gut permeability can lead to translocation of pathogenic bacteria into the bloodstream and increased immune‐mediated toxicities during immunotherapy [113]. Improved intestinal permeability and tight junction protein expression were associated with a better response to anti‐PD‐1 therapy [114]. Similarly, increased stress or the application of dexamethasone, which is not allowed during immunotherapy because it blunts the response, increased gastrointestinal (GI) permeability [115].

## 8 Cannabinoids Influencing the Gut Microbiome

There is growing evidence that cannabis use can change the composition of the gut microbiome. The gut microbiota of mice treated with THC for an extended period of time increased the relative abundance of the *Akkermansia muciniphila* bacterium, which is associated with better intestinal barrier function and metabolic health. On the other hand, a higher number of Bacteroides species has been associated with the use of cannabis in the human gut microbiota. This could be related to metabolic problems and intestinal inflammation. These results imply that cannabis use may have an impact on the composition of the gut microbiota, which may have an impact on gut health and other physiological processes that the gut microbiome regulates [116].

**FIGURE 2:**
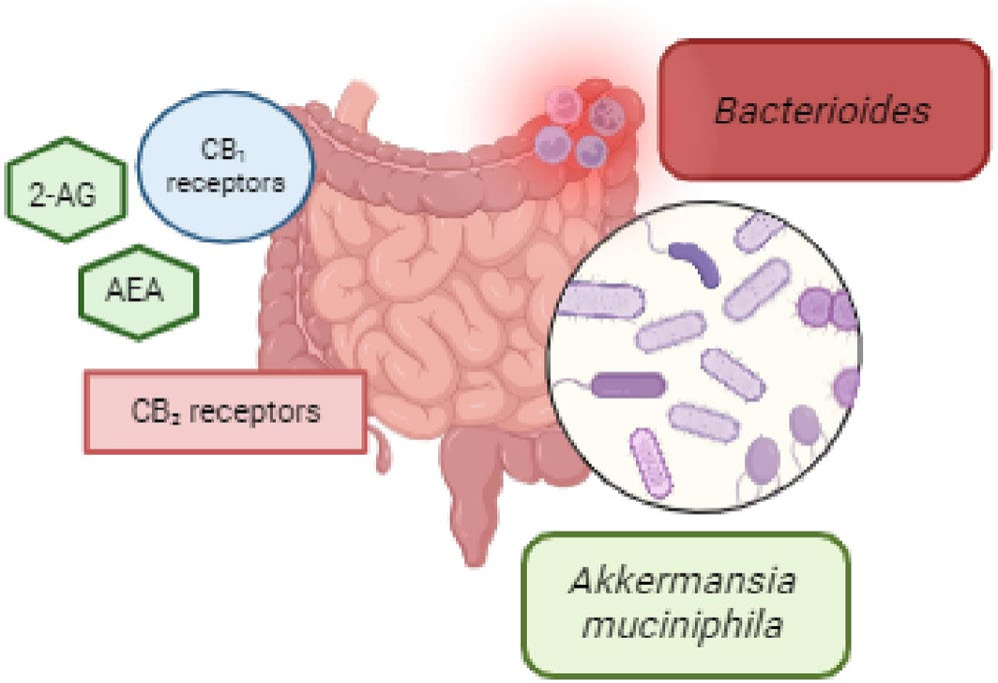
Relationship between cannabinoids and gut microbiome. This figure illustrates how cannabinoids influence the composition of the intestinal microbiota, promoting beneficial bacterial strains such as *Akkermansia muciniphila*. Changes in the intestinal microbiota can regulate the activity of the endocannabinoid system by modulating CB_1_ and CB_2_ receptors in the gastrointestinal tract. A higher number of *Bacteroides* species is related to metabolic problems and intestinal inflammation.

Although microbiome strains can induce the expression of both CB_1_ and CB_2_ receptors, the other way is also true, as selective antagonism of CB_1_ receptors resulted in changes in the microbiome, particularly *A. muciniphila* , with a decrease in Lachnospiraceae and Erysipelotrichaceae [117]. Similarly to *Akkermansia*, Lachnospiraceae were also shown to be significantly more abundant in responders to anti‐PD‐1 immunotherapy in hepatobiliary cancers, compared to non‐responders [118] (Figure 2).

Another study reported that the treatment of mice with CBD‐enriched cannabis extracts showed an increase in the relative abundance of the probiotic *A. muciniphila* , as well as an increase in pro‐inflammatory cytokines and chemokines in colon tissue, along with a decreased expression of Muc2 (a gene associated with intestinal integrity) [119].

## 9 Conclusion

The endocannabinoid system is widely distributed in both normal and cancer cells, including the tumor microenvironment and cancer cells themselves. Most preclinical studies have shown that cannabinoids induce a reduction in tumor growth. It was shown that a positive relationship exists between CB_2_ receptors and survival in skin melanoma, while higher levels of CB_2_ found in carcinogen‐exposed skin led to increased antitumor activity when receptors were activated.

Further complexity lies in the fact that the effect of cannabinoids appears to be dose‐dependent and bimodal, with lower concentrations increasing cancer proliferation, whereas higher concentrations induce apoptosis in cancer cell lines. Furthermore, there is a significant relationship between the state of the cancer microenvironment, the gut microbiome, and the expression of cannabinoid receptors that could explain the lack of consistency between studies. It has become apparent that cannabinoids are not recommended in patients receiving immunotherapy, particularly when patients are in an advanced metastatic stage of the disease. However, this does not negate the potential use of cannabinoids as an adjunct when patients are qualified for targeted therapies such as BRAF inhibitors and MEK inhibitors. However, although the evidence supporting this combination therapy is still in the early stages, several studies suggest that cannabinoids might improve the efficacy of targeted treatments by influencing the immune response, modulating the tumor microenvironment, or mitigating some adverse effects of these therapies. Therefore, randomized placebo‐controlled clinical trials with accurate doses of cannabinoids are required to substantiate the findings.

## Author Contributions

All authors contributed and reviewed the manuscript.

## Conflicts of Interest

The authors declare no conflicts of interest.

## Data Availability

The data supporting the findings of this study are available within the article and will be available following the publication of the manuscript.
